# Precision of time-resolved near-infrared spectroscopy-based measurements of cerebral oxygenation in preterm infants

**DOI:** 10.1117/1.NPh.8.4.045001

**Published:** 2021-10-22

**Authors:** Alexander Avian, Christian Mattersberger, Lukas Schober, Johann Martensen, Martin Wolf, Wataru Kamo, Gerhard Pichler, Bernhard Schwaberger, Berndt Urlesberger

**Affiliations:** a Medical University of Graz, Institute for Medical Informatics, Statistics, and Documentation, Graz, Austria; b Medical University of Graz, Division of Neonatology, Graz, Austria; c Medical University of Graz, Research Unit for Neonatal Macro- and Microcirculation, Graz, Austria; d Medical University of Graz, Research Unit for Cerebral Development and Oximetry Research, Graz, Austria; e University of Zurich, University Hospital Zurich, Department of Neonatology, Biomedical Optics Research Laboratory, Zurich, Switzerland; f Hamamatsu Photonics K.K., Systems Division, Hamamatsu, Japan

**Keywords:** time-resolved near-infrared spectroscopy, cerebral oxygenation, precision, preterm infants

## Abstract

**Significance:** Time-resolved near-infrared spectroscopy (t-NIRS) is a new technology; at the moment, data on its precision in preterm infants are rare.

**Aim:** Therefore, the aim of this study was to analyze the precision of t-NIRS-based measurements of the cerebral oxygenation in preterm infants.

**Approach:** In 70 neonates [age: 4.7±2.0 days, sex (f/m): 33/37], cerebral oxygenation ($t\text{-}{\mathrm{rSO}}_{2}$) was measured with an optode placed over the left frontotemporal lobe on the head, measurement duration was 1 min, and a reapplication was done for four further times (five applications).

**Results:** Overall mean for $t\text{-}{\mathrm{rSO}}_{2}$ values was 62.2%±4.1%. We found a within-patient variation for $t\text{-}{\mathrm{rSO}}_{2}$ of 2.6%. Furthermore, 95% of all observed values were within a range of ±5% from the mean when looking on several reapplications and ±2% when looking within one application. Most of the variation in $t\text{-}{\mathrm{rSO}}_{2}$ (60.4%) contributed to differences between patients. The remaining 39.6% of the variation was due to measurement errors and real changes of the measured signal within the neonates.

**Conclusions:** Since within-patient variation of $t\text{-}{\mathrm{rSO}}_{2}$ measures were below a clinical meaningful threshold of 5%, the measurement can be denoted as precise.

## 1 Introduction

In the fetal to neonatal transition, cerebral oxygen saturation may not increase appropriately. Especially, preterm neonates are affected by hypoxia, which may lead to perinatal brain injury.^1^^–^^3^ As a consequence, the risk of perinatal death, cerebral palsy, cognitive, behavioral, and memory problems is increased.^4^^–^^7^

Optical methods such as near-infrared spectroscopy (NIRS) enable non-invasive monitoring of cerebral oxygenation. NIRS has been used in research in both preterm and term neonates. Typical changes in the cerebral oxygenation in the first minutes after birth as well as differences in these changes according to need of respiratory support were evaluated.^8^^–^^10^ NIRS has also been successfully used to guide respiratory support and supplemental oxygen to reduce the burden of cerebral hypoxia during immediate transition and resuscitation after birth.^11^ Furthermore, differences between neonates small for gestational age and appropriate for gestational age neonates and changes of cerebral oxygenation due to painful interventions have been observed.^12^^,^^13^

Given the importance of cerebral oxygenation measurements, the reproducibility of this measurement is crucial. Although Szczapa et al.^14^ found substantially different precisions in cerebral oxygenation, Hyttel-Sorensen et al.^15^ were reporting quite similar precision values for non-cerebral regional tissue hemoglobin saturation measurements in three out of four investigated devices.

All of the studies mentioned above were conducted with NIRS, using spatially resolved spectroscopy, a type of continuous wave technique, which allows the continuous measurement of the oxygenated and deoxygenated hemoglobin concentration times the scattering coefficient.^16^ When calculating the regional cerebral oxygenation (${\mathrm{rSO}}_{2}$) (also called tissue oxygenation index), the scattering coefficient cancels out.

Time-resolved near-infrared spectroscopy (t-NIRS) emits extremely short pulses of light (duration: between few tens of picoseconds to ∼1  ns) into the tissue and measures the time-of-flight of these photons through the tissue. The distribution of the time-of-flights enables to determine the absolute values of the scattering and absorption coefficients and from the latter, the tissue oxygen saturation ($t\text{-}{\mathrm{rSO}}_{2}$).^17^ For a different device (Babylux), it has been shown recently that t-NIRS technology achieved an improved intrasubject variability for cerebral oxygenation measurements when compared with spatially resolved spectroscopy.^18^ The primary aim of this study was to estimate precision of cerebral $t\text{-}{\mathrm{rSO}}_{2}$ measurements for a new time-resolved NIRS device, the tNIRS-1 (Hamamatsu Photonics K.K., Hamamatsu, Japan). Furthermore, the secondary aim was to investigate the precision of ${\mathrm{SpO}}_{2}$, heart rate (HR), t-HB, $t\text{-}O_{2}\mathrm{Hb}$, and t-HHb measures.

## 2 Methods

The present prospective observational study complies with all institutional guidelines related to patient confidentiality and research ethics, including Institutional Review Board approval (EC-Numbers: 29-351 ex 16/17). It was carried out at the Medical University Hospital, Graz, Austria. As the study focused on precision of the device, the protocol included only preterm infants (gestational age<37 weeks) in a stable clinical situation. Hence, exclusion criteria were: need of any respiratory support or supplemental oxygen, compromised cardio-circulation in need of treatment with inotropes, severe cerebral injury [e.g., intracranial hemorrhage > grade II, stroke, hypoxic-ischemic encephalopathy > grade 1], and the presence of major congenital malformations. To ensure that preterm infants were within normal hemoglobin ranges, only infants who had a venipuncture with determination of hemoglobin not longer than 5 days ago were included. Furthermore, only infants with written informed consent signed by their parent or legal guardian were eligible for inclusion.

### 2.1 Device

For this study, the tNIRS-1 (Hamamatsu Photonics K.K., Hamamatsu, Japan) was used. tNIRS-1 employs semiconductor laser diodes (LD) with three different wavelengths of 755, 816, and 850 nm as light sources. The LDs emit pulses with a repetition of 9 MHz for each wavelength through an optode consisting of an optical fiber bundle and a right-angle prism. Full-width at half-maximum of the instrument response function (IRF) is ∼1.5  ns.^19^ Transmitted light through the human tissue is collected by another optode and detected by a cooled multipixel photon counter. The temporal distribution of the time of flights (TOF) of detected photons is recorded by a time-to-digital convertor for the three wavelengths sequentially. tNIRS-1 automatically attenuates the light so that photon counting rate is between 60 and 210 kcps. In this range, the TOF distributions are virtually undistorted and have a sufficient signal-to-noise ratio.^20^ The photon diffusion equation is fitted into TOF histograms^21^ to determine both the absorption and scattering coefficients by the non-linear least square method.

The IRF is updated by trained staff every day to minimize effects of fluctuation of the system characteristics. The IRF is recorded using a custom fiber holder including attenuation layer and diffuser slab, which enables to illuminate all waveguide modes of the detection fiber bundle.

Oxygenated hemoglobin ($O_{2}\mathrm{Hb}$) and deoxygenated hemoglobin (HHb) are then calculated from the absorption coefficients by ordinary NIRS methods.^22^ Subsequently, cerebral oxygenation ($t\text{-}{\mathrm{rSO}}_{2}$) and total hemoglobin (${\mathrm{Hb}}_{\mathrm{tot}}$) are derived by ($t\text{-}{\mathrm{rSO}}_{2}=O_{2}\mathrm{Hb}/(O_{2}\mathrm{Hb}+\mathrm{HHb})$ and (${\mathrm{Hb}}_{\mathrm{tot}}=O_{2}\mathrm{Hb}+\mathrm{HHb}$), respectively.

### 2.2 Measurement Protocol

In each neonate, $t\text{-}{\mathrm{rSO}}_{2}$ was measured five times (five reapplications) for 1-min each. $t\text{-}{\mathrm{rSO}}_{2}$ was measured using two optodes with source-detection separation of 3 cm, fixated on a probe pad, which was placed over the left frontotemporal lobe with a biocompatible adhesive tape. Between measurements, the probe pad was removed and reapplicated at approximately the same location without marking. $O_{2}\mathrm{Hb}$, HHb, and $t\text{-}{\mathrm{rSO}}_{2}$ were recorded. Peripheral arterial oxygenation (${\mathrm{SpO}}_{2}$) and HR were measured with pulse oximeter at the right hand or wrist (IntelliVue MP30/X2 Monitor, Philips, Netherlands) using Masimo Signal Extraction Technology (Masimo Corporation, Irvine, CA, USA). All variables were stored continuously in a multichannel system “alpha-trace digital MM” (Best Medical Systems, Vienna, Austria) for subsequent analyses. One measurement cycle for a $t\text{-}{\mathrm{rSO}}_{2}$ value needs 5 s. Total hemoglobin (${\mathrm{Hb}}_{\mathrm{tot}}$) was calculated for each 5 s period.

### 2.3 Statistical Analysis

Sample size calculation was performed using 95% confidence intervals for the precision of measurement of cerebral tissue oxygenation. The precision of measurement of cerebral tissue oxygenation can be defined as the variation of consecutive measurements. In their study, Sorensen and Greisen^23^ observed a within infant variation of 5.2% when repositioning the optodes. In line with this result, a difference of 5% was defined as clinically relevant difference. Therefore, our sample size calculation was based on an upper limit of the 95% confidence interval of the precision of 5%. According to the algorithm of Hopkins,^24^^,^^25^ 65 neonates have to be included to obtain an upper limit of the 95% confidence interval of 5% when the precision is 4.5% and five reapplications of the optodes are performed. Considering a dropout rate of 10%, 73 neonates had to be included.

For the primary analysis for calculating the precision of $t\text{-}{\mathrm{rSO}}_{2}$ measurement, a variance component analysis was conducted to quantify the factors influencing the variability in $t\text{-}{\mathrm{rSO}}_{2}$ values. Therefore, a mixed effects model with random effect (patient), fixed effects (reapplication, single application, interaction of reapplication × single application), and $t\text{-}{\mathrm{rSO}}_{2}$ as a dependent variable was used. A minimum norm quadratic unbiased estimation model with unit *a priori* values for the ratios of the variance components to the residuals variance and the residual itself was chosen. Resulting variance components were transformed to percentages of the whole variance. Furthermore, the square root of the variance components was calculated to obtain a parameter to quantify the variation. 95% confidence intervals for variance components were calculated by the bootstrapping technique. This bootstrapping algorithm draws 1000 samples using simple bootstrap resampling. 95% confidence intervals were calculated using the percentile method.

To describe the precision of the measurement, the within-patient variation was reported. For calculating a descriptive measure of the observed variation of the measurement, the deviation of the observed $t\text{-}{\mathrm{rSO}}_{2}$ values to the individual mean was calculated ($t\text{-}{\mathrm{rSO}}_{2}$). 2.5% and 97.5% percentile scores for these deviations with associated 95% confidence intervals were calculated by the bootstrapping technique. This bootstrapping algorithm draws 1000 samples using simple bootstrap resampling. 95% confidence intervals were calculated using the percentile method. The same approach was used to analyze secondary outcomes (${\mathrm{SpO}}_{2}$, HR, t-HB, $t\text{-}O_{2}\mathrm{Hb}$, and t-HHb). Analyses were conducted with no imputation for any missing data. In the primary outcome ($t\text{-}{\mathrm{rSO}}_{2}$), 2.4% missing values occurred. For the secondary parameters, the rate of missing values ranged between 1.6% and 2.8% with an overall rate of missing values of 2.4%. Statistical analysis was performed using IBM SPSS Statistics 24.0.0 (SPSS Inc., IBM Company, Chicago, IL, USA).

## 3 Results

**Fig. 1:**
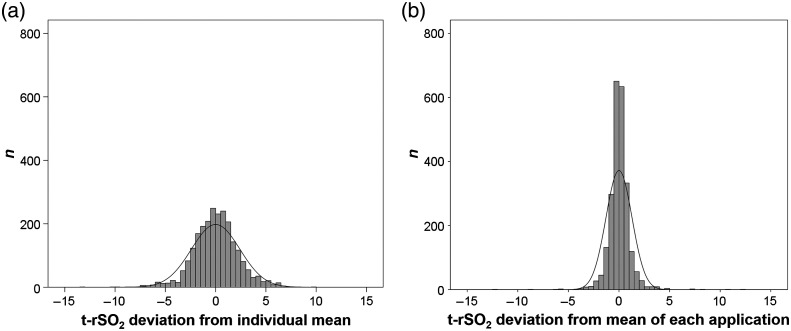
Distribution of the deviation of each $t\text{-}{\mathrm{rSO}}_{2}$ measurement from (a) the individual overall mean and (b) mean of each application.

Between October 2017 and August 2018, 73 preterm infants were included into the study. Three infants (4%) were excluded due to incomplete data. The descriptive data of remaining 70 infants are displayed in Table 1. Conducting five reapplications, each resulting in at least seven NIRS values per 1 min measurement period, resulted in maximum of 2450 possible values for each parameter. For $t\text{-}{\mathrm{rSO}}_{2}$, 59 missing values were observed. The grand mean for the remaining 2391 $t\text{-}{\mathrm{rSO}}_{2}$ values was 62.2%±4.1%. Looking on each reapplication separately, the mean values for all measured values were 62.9%±4.0% (application 1), 62.0%±4.2% (application 2), 62.2%±4.4% (application 3), 61.7%±4.0% (application 4), and 62.0%±4.0% (application 5). 95% of the measured $t\text{-}{\mathrm{rSO}}_{2}$ values varied in a range from −4.96 to 4.80 percent points from the overall individual mean. Taking the deviation of the mean of each single application for each patient, this range reduced to −1.96 to 2.05 (Table 2 and Fig. 1). In the primary outcome, $t\text{-}{\mathrm{rSO}}_{2}$ variance components that were attributed to differences between patients represented 60.4% of the total variance. Further, 28.3% were assigned to differences between reapplications, 1.3% to differences within one single application, and 10.1% to different changes within applications (Table 3). The precision of the measurement defined as within variation in $t\text{-}{\mathrm{rSO}}_{2}$ was 2.6%.

**Table 1:** Demographic and clinical characteristics of the included neonates [mean ± SD, median (IQR) or n] (APGAR: method to evaluate health of newborn by rating their appearance, pulse, grimace, activity, and respiration).

Age (days)	4.7 ± 2.0
Sex (f/m)	33/37
Gestational age (week)	33.4 ± 1.7
Weight (birth) (g)	1931 ± 398
Weight (measurement) (g)	1876 ± 388
APGAR1	8 (7 to 9)
APGAR5	9 (8 to 10)
APGAR10	9 (9 to 10)
$t\text{-}{\mathrm{rSO}}_{2}$ (%)	62.2 ± 4.1
${\mathrm{SpO}}_{2}$ (%)	95.9 ± 2.8
HR (bpm)	140.3 ± 14.8
t-Hb (μmol)	45.2 ± 8.5
$t\text{-}O_{2}\mathrm{Hb}$ (μmol)	28.2 ± 5.9
t-HHb (μmol)	17.0 ± 3.4

**Table 2:** Observed deviation of each measurement from the individual overall mean and mean of each application.

	Deviation from			
	Overall mean		Mean of each single application	
	2.5% percentile (95% CI)	97.5% percentile (95% CI)	2.5% percentile (95% CI)	97.5% percentile (95% CI)
$t\text{-}{\mathrm{rSO}}_{2}$ (%)	−4.96 (−5.44 to −4.29)	4.80 (4.38 to 5.10)	−1.96 (−2.16 to −1.72)	2.05 (1.85 to 2.31)
${\mathrm{SpO}}_{2}$ (%)	−3.77 (−4.44 to −3.33)	3.04 (2.86 to 3.18)	−2.17 (−2.41 to −1.97)	2.04 (1.93 to 2.21)
HR (bpm)	−11.89 (−13.11 to −11.05)	15.10 (13.40 to 16.49)	−8.43 (−9.29 to −7.00)	7.96 (7.25 to 8.57)
t-Hb (μmol)	−7.43 (−7.99 to −6.69)	8.91 (7.43 to 9.49)	0.92 (−1.06 to −0.85)	0.93 (0.86 to 1.01)
$t\text{-}O_{2}\mathrm{Hb}$ (μmol)	−5.32 (−5.60 to −4.98)	5.64 (5.14 to 6.03)	−1.27 (−1.49 to −1.19)	1.29 (1.14 to 1.43)
t-HHb (μmol)	−2.93 (−3.04 to −2.83)	3.48 (3.26 to 3.91)	−0.67 (−0.73 to −0.60)	0.69 (0.63 to 0.72)

**Table 3:** Absolute and relative variance components of patients, reapplication, and single application with 95% confidence intervals. Results are based on a mixed effects model with random effect (patient) and fixed effects (reapplication and single application). Resulting variance components were transferred to percentages of the whole variance to show the proportion of the variance that is due to (1) the differences between patients, (2) reapplications, (3) differences within a single application, and (4) differences in the changes within a single application between reapplications (interaction).

		Variance components			
		Patient	Reapplication	Single application	Interaction of reapplication × single application
$t\text{-}{\mathrm{rSO}}_{2}$ (%)	Value	10.5 (7.1 to 14.1)	4.9 (3.5 to 6.4)	0.2 (0.0 to 0.6)	1.8 (0.9 to 2.9)
	%	60. 4 (58.8 to 62.4)	28.3 (26.8 to 30.5)	1.3 (0.0 to 2.6)	10.1 (7.5 to 12.5)
${\mathrm{SpO}}_{2}$ (%)	Value	4.9 (3.0 to 7.1)	1.9 (1.3 to 2.4)	0.0 (0.0 to 0.0)	1.2 (1.0 to 1.4)
	%	61.8 (56.7 to 65.3)	23.2 (21.8 to 25.2)	0.0 (0.0 to 0.6)	15.0 (13.1 to 18.1)
HR (bpm)	Value	165.0 (111.4 to 219.9)	37.9 (23.2 to 56.1)	0.0 (0.0 to 1.3)	19.7 (12.9 to 28.0)
	%	74.1 (72.4 to 75.6)	17.0 (15.7 to 18.4)	0.0 (0.0 to 0.6)	8.8 (8.6 to 9.2)
t-HB (μmol)	Value	56.0 (34.6 to 79.8)	16.5 (10.6 to 23.6)	0.0 (0.0 to 0.0)	0.3 (0.2 to 0.3)
	%	76.9 (76.0 to 77.1)	22.7 (22.5 to 23.6)	0.0 (0.0 to 0.0)	0.4 (0.3 to 0.4)
$t\text{-}O_{2}\mathrm{Hb}$ (μmol)	Value	26.2 (15.3 to 38.4)	8.1 (5.7 to 10.9)	0.0 (0.0 to 0.0)	0.5 (0.4 to 0.6)
	%	75.4 (71.8 to 77.0)	23.2 (21.8 to 26.6)	0.1 (0.0 to 0.1)	1.3 (1.1 to 1.7)
t-HHb (μmol)	Value	8.9 (5.7 to 12.0)	2.9 (1.9 to 4.0)	0.0 (0.0 to 0.0)	0.1 (0.1 to 0.2)
	%	74.5 (73.5 to 74.7)	24.4 (24.1 to 25.3)	0.0 (0.0 to 0.1)	1.1 (1.0 to 1.3)

Looking on each application separately, variance components that were assigned to within-patient variations represented 4.3% to 16.1% of the total variance of the respective application. The resulting precision of each application was 1.3% (application 1), 1.7% (application 2), 0.9% (application 3), 1.5% (application 4), and 1.4% (application 5).

Variance components for the secondary outcome parameters (${\mathrm{SpO}}_{2}$, HR, t-HB, $t\text{-}O_{2}\mathrm{Hb}$, and t-HHb) that could be assigned to differences between patients represented 61.8% to 75.4% of the total variance. Again, the part assigned to the reapplication was the second largest (17.0% to 32.6%). Precision for the secondary outcome was 2.2% for ${\mathrm{SpO}}_{2}$, 12.8 bpm for HR, 7.5 [μmol] for t-Hb, 5.1 [μmol] for $t\text{-}O_{2}\mathrm{Hb}$, and 3.0 [μmol] for t-HHb. Deviations from the overall mean and the mean of each application for each secondary outcome parameters can be seen in Table 3.

## 4 Discussion

In our study, we found a within patient precision for $t\text{-}{\mathrm{rSO}}_{2}$ of 2.6%. This value is below the threshold of 3% mentioned by Greisen et al.^26^ and below reported precision in other studies. Kleiser et al.^27^ summarized that typically precision in the measurement of cerebral oxygenation is ∼5%. Furthermore, in our study, 95% of all observed values were within a range of ±5% from the mean when looking on several reapplications and ±2% when looking within one application. Most of the variation in $t\text{-}{\mathrm{rSO}}_{2}$ (∼2/3) stemmed from differences between patients. The remaining 1/3 of the variation was due to measurement errors and real changes of the measured signal.

Comparison of results of this study to previously reported values on the precision of NIRS measurements shows that this study is within the range of reported values. Previously reported precision as within infant variation for ${\mathrm{rSO}}_{2}$ was between 1.7% and 8.2%.^18^^,^^22^^,^^28^^,^^29^ The high variability of reported precision values has to be assigned to the fact that different devices were used, and different source–detector distances and measurement protocols were used. Szczapa et al.^14^ was able to show that using two different measurement devices resulted in substantially different variations of the measurement (e.g., ±7.6 versus ±3.9), and therefore differences in the precision of these two devices. It has to be pointed out that all the above studies analyzed devices using continuous wave technology, which is different from the technique of this study.

### 4.1 Reapplication and Sources for Variations in the Recorder Signal

Changes in the recorded signal due to the reapplication can reflect real differences in the measured signal or measurement errors derived from the reapplication. Real differences in the measured signal can be due to changes in the neuronal activity over time (e.g., arousal due to the replacement of the optodes), hemodynamic fluctuations, or regional differences in neuronal activity within the area, where the optodes were reapplied. Therefore, there might be differences in local oxygen delivery and consumption.

There might be several sources for measurement errors. First, an important source of measurement errors is tissue inhomogeneity. Inhomogeneity is caused by the presence of hair, moles, blood vessels dirt, and gyri. The different degree of inhomogeneity influences the amount of measurement errors when reapplications are not done at the exact same point. Arri et al.^30^ showed that with increasing tissue homogeneity, the precision (within standard deviation of the measurement) of ${\mathrm{StO}}_{2}$ measures improved from 11% to 2% and the precision of t-Hb from 16.4 to 3.5  μM. Second, another source of possible measurement errors is the difference in cerebro-spinal fluid space geometry. Consequently, measurements in different neonates are faced with partly different geometries.^23^ Therefore, within variations in the measured signal may be caused by real changes in the cerebral blood flow, changes in cerebral oxygen delivery, movement artifacts, or different optode locations, resulting in distortions of the geometry and finally to measurement errors. Hyttel-Sorensen et al.^15^ concluded that imprecision of the measurement is mainly due to optical heterogeneity and therefore does not reflect spontaneous fluctuations. In contrast to this conclusion, Kleiser et al.^27^ were able to substantially improve precision by adding ${\mathrm{SpO}}_{2}$ measurements of the right arm and continuous ${\mathrm{StO}}_{2}$ measurements on the head and thus quantifying spontaneous systematic hemodynamic fluctuations. This is important since the precision can only be determined correctly, if it can be assumed that the parameter to be measured is actually constant. This is, of course, not the case in living tissue.

Other aspects that influence the precision of t-NIRS are the absolute $t\text{-}{\mathrm{rSO}}_{2}$ value and total photon counts. Giovannella et al.^31^ showed the precision of t-NIRS measurements increased with increasing the number of total photon counts and increasing absolute $t\text{-}{\mathrm{rSO}}_{2}$ values. Since these parameters were not manipulated within our study and most of the neonates’ $t\text{-}{\mathrm{rSO}}_{2}$ values are within a small range (IQR of all measures: 59.8 64.8), the influence of these parameters could not be analyzed in this study. Nevertheless, when measuring low $t\text{-}{\mathrm{rSO}}_{2}$ values, one should keep in mind that precision may be worse.

The amount of variation, which may be caused either by the measurement device or by real changes within the neonate (variance component of the within-patient variation), was reported to be between 12% and 35%.^28^^,^^29^^,^^32^ In our study, this proportion was higher (39.6%; sum of differences between applications, differences within one application, and different changes within applications). Possible reasons for our higher within-patient variation are (1) a more homogeneous patient group and therefore less between-patient variation, (2) different time intervals and therefore different amounts of real changes, and/or (3) different precisions of the tNIRS-1 compared to other measurement devices used in other publications.

### 4.2 Repeated Measurements

As reported by Sorensen and Greisen,^23^ repeated measurements of NIRS parameters lead to smaller number of patients needed for clinical research. According to our study, 39 neonates in each group are needed to detect a difference of 3% (1−β: 0.90, α: 0.05) if the population’s standard deviation is the same we have observed in our first application (SD: 4.0%). Having five applications in each neonate reduces the number of required neonates to 26 in each group. Beside the importance of repeated measurements in NIRS detection for research purposes, this is also an issue for daily clinical routine work. Increasing the number of measurements has been reported to increase reliability substantially.^23^ In our study, about 70% of the within variation was due to the reapplication and therefore reflect either real patient related changes or changes of the values due to the reapplication of the optodes. Although this big proportion of variation due to the reapplications decreases precision, it is important to mention that from a viewpoint of validity, these reapplications are important. By applying the optodes only once, values influenced by the presence of hair, moles, and other superficial features will be missed. One disadvantage of the current t-NIRS approach is that it measures only at one distance and hence, the influence of such superficial features can neither be detected nor reduced. If there were several source–detector pairs, outliers due to superficial features could be identified and removed. In addition, the $t\text{-}{\mathrm{rSO}}_{2}$ values also include a substantial portion that does not reflect the brain but the superficial tissue (e.g., skin and skull). For continuous wave and frequency domain instruments, it has been shown that by measuring at several distances, it is possible to remove this influence.^33^ In principle, it is also possible to employ the multidistance approach for the time-domain approach. Furthermore, it has been shown that the distribution of TOF enables to increase the sensitivity to deep tissue layers.^34^

An important point of discussion in this context is physiological changes that are patient related. For determining the precision, the prerequisite is that the parameter to be measured does not change. Even in a stable infant, this is not the case. This means that the measurement error is overestimated, because part of the variability reflects real physiological changes. Thus in reality the precision is better. In addition, from a clinical point of view, measurements of cerebral ${\mathrm{rSO}}_{2}$ are more important in unstable infants, where ${\mathrm{rSO}}_{2}$ is expected to change a lot. The clinical value of ${\mathrm{rSO}}_{2}$ is to assess either significant parameter changes and/or to reduce the burden of hypoxia/hyperoxia. Therefore, in clinical situations with expected rapid and high changes, it does not make sense to apply the method of repeated measurements.

### 4.3 Improving Precision

Precision of measurement can be improved by introducing quality criteria for the measurement. Kleiser et al. showed that precision in $t\text{-}{\mathrm{rSO}}_{2}$ values increased without any further quality criteria from 2.64% (within-patient variation) to 2.02% just by excluding patients with motion artifacts. Excluding further patients with unstable ${\mathrm{SpO}}_{2}$ finally improved precision to 1.85%. Doing so, the number of analyzed neonates decreased from n=33, or n=35 (depending on the region of interest), to n=24, respectively.^27^ As a consequence of reducing the within-patient variation, the proportion of the variance component caused by measurement errors decreases. Pichler et al.^32^ showed that after eliminating implausible values the proportion of within patient variance component decreased from 46.6% to 35%. In our study, we decided not to introduce quality criteria that can only be evaluated after the measurement is completed. By doing so, we still included values that might even be due to measurement errors (see Fig. S1 in the Supplementary Material). By doing that, we accepted precision values that were inferior to precision values obtained with optimal measurements. We proceeded like this, because these values reflect precision of clinical routine work more accurately than optimized measurements. However, the observed precision was still high.

Nevertheless, a further way to improve precision is to consider the optical heterogeneity. Using more measurements to improve precision not necessarily results in the use more time points in this case. Hyttel-Sorensen et al.^15^ suggested to use two measurement channels at the same time and consecutively to calculate the mean of these two values. Each of these two measurements will be affected by optical heterogeneity in a different amount and thus the total effect of optical heterogeneity will be reduced.

### 4.4 Limitation and Conclusion

Although the tested instrument provides a high precision, there are some limitations. Currently, the instrument only provides one ${\mathrm{rSO}}_{2}$ value every 5 s, which is slower than other instruments.

In conclusion, we found the precision of $t\text{-}{\mathrm{rSO}}_{2}$ measurements to be 2.6%, when using five reapplications. The amount of precision should be considered when interpreting the results of a measurement. Assuming a clinically relevant lower threshold of 60%, three out of 100 measurements would be below this threshold when the display shows 65%, and 23 out of 100 when the display shows 62%. Using a slightly less precision measurement with a precision of 3% would increase these numbers to 5/100 and 26/100 and with a precision of 5% to 16/100 and 35/100.

## Code, Data, and Materials Availability

Data underlying the results presented in this paper are not publicly available at this time but may be obtained from the authors upon reasonable request.
