# Supplementary material for: Precision of time-resolved near-infrared spectroscopy-based measurements of cerebral oxygenation in preterm infants
Source: Neurophotonics. 2021 Oct 22;8(4):045001. doi: 10.1117/1.NPh.8.4.045001 (PMC8536243; doi:10.1117/1.NPh.8.4.045001)
Supplement: Supplementary file 1 [file NPh_008_045001_SD001.pdf]

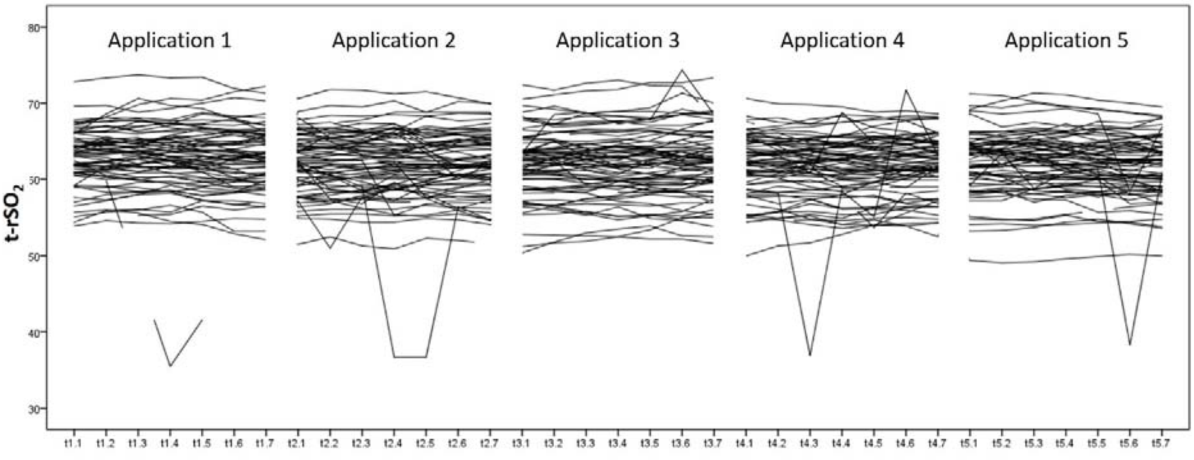

Fig. S1 Individual courses of t-rSO_2. Values that are far away from the other that might be outliners due to measurements errors were not deleted to get results that are near to real life measurements.
